# Biochemical characterization of paralyzed flagellum proteins A (PflA) and B (PflB) from *Helicobacter pylori* flagellar motor

**DOI:** 10.1042/BSR20240692

**Published:** 2024-09-10

**Authors:** Xiaotian Zhou, Muhammad F. Khan, Yue Xin, Kar L. Chan, Anna Roujeinikova

**Affiliations:** 1 Department of Microbiology, Biomedicine Discovery Institute, Monash University, Melbourne, Victoria 3800, Australia; 2 Department of Biochemistry and Molecular Biology, Monash University, Melbourne, Victoria 3800, Australia

**Keywords:** bacterial flagellar motor, Helicobacter pylori, microbial motility, protein purification, protein refolding

## Abstract

Motility by means of flagella plays an important role in the persistent colonization of *Helicobacter pylori* in the human stomach. The *H. pylori* flagellar motor has a complex structure that includes a periplasmic scaffold, the components of which are still being identified. Here, we report the isolation and characterization of the soluble forms of two putative essential *H. pylori* motor scaffold components, proteins PflA and PflB. We developed an on-column refolding procedure, overcoming the challenge of inclusion body formation in *Escherichia coli*. We employed mild detergent sarkosyl to enhance protein recovery and n-dodecyl-N,N-dimethylamine-N-oxide (LDAO)-containing buffers to achieve optimal solubility and monodispersity. In addition, we showed that PflA lacking the β-rich N-terminal domain is expressed in a soluble form, and behaves as a monodisperse monomer in solution. The methods for producing the soluble, folded forms of *H. pylori* PflA and PflB established in this work will facilitate future biophysical and structural studies aimed at deciphering their location and their function within the flagellar motor.

## Introduction

*Helicobacter pylori* is a Gram-negative carcinogenic bacterium that colonizes the gastric epithelium in nearly half of the world’s population [1]. Prior to the discovery of *H. pylori*, the stomach was considered to be a sterile organ [2,3], and the ability of *H. pylori* to thrive in this extremely acidic environment is remarkable. The pathological changes in the epithelial layer, associated with the *H. pylori* infection, are driven in large part by the secretion of *H. pylori* toxins [4–6], but central to disease development is the ability of *H. pylori* to persist in the stomach long term through adaptations [7] and evasion of the host immune response. The bacterium deploys an array of tactics to avoid being killed by the host immune system: it employs glycosylation to shield its surface proteins [8]; neutralizes reactive oxygen species generated by macrophages [9]; and produces enzymes capable of degrading innate immune peptides [10]. In addition, *H. pylori* employs chemotaxis (directed flagella-driven motility) to avoid elimination by host complement [11].

The latter discovery has increased the appreciation of the significance of *H. pylori* motility in pathogenesis, adding to the knowledge that *H. pylori* needs to be motile to be able to colonize the host and to achieve full infection levels [12], and that it uses chemotaxis to seek out nutrients [13,14]. However, we are just beginning to understand how the molecular nanomachine that drives the rotation of the flagellum – the *H. pylori* flagellar motor – functions, in terms of its structure, components and their individual roles.

Although the structure and function of the power-generating (stator) units of the flagellar motor are highly conserved in *H. pylori* and *Escherichia coli* [15–17], electron cryotomography visualization of the motor in whole *H. pylori* cells [18–20] revealed that it is much wider and significantly more complex than the flagellar motor in *E. coli*, because it has an additional periplasmic scaffold. This is significant because similarly complex, but structurally distinct periplasmic scaffolds have been found in polar flagellar motors in many other bacteria [21,22], and their molecular composition and function have been the subject of extensive recent research. The studies in *H. pylori* have identified the first components of the periplasmic scaffold, the stator-associated FliL [19,23] and the homologs of type IV pili proteins PilO, PilM and PilN [20,24]. In addition, the function of the *H. pylori* peptidoglycan-associated lipoprotein (Pal) has been linked to the motor, although it is not yet known if *H. pylori* Pal forms part of the motor scaffold [25].

The identity and the role of the rest of the periplasmic scaffold of the *H. pylori* flagellar motor remains to be established. However, we noticed that this bacterium possesses genes encoding homologs of paralyzed flagellum proteins A (PflA) and B (PflB) found in the scaffold of the motor in the closely related bacterium *Campylobacter jejuni* [26]. The *C. jejuni pflA* gene was discovered next to the gene encoding a chemotaxis receptor by Yao et al. who observed that the respective mutation resulted in a flagellated but non-motile phenotype [27]. The ability of the *C. jejuni pflA* mutant cells to adhere to, and invade, human epithelial cells was significantly reduced compared with the wild-type [27]. Subsequently, Gao et al. identified a gene, located elsewhere on the *C. jejuni* chromosome, that encodes a protein that interacts with PflA, and the loss of which resulted in the similar non-invasive, paralyzed flagellum phenotype [28]. The protein, termed PflB, was shown to localize to the cell poles, suggesting a role associated with the function of the flagellar motor. The subsequent electron cryotomography studies of the *pflA* and *pflB* mutants of *C. jejuni* [21] suggested that PflA and PflB are integral structural components of the periplasmic scaffold of its flagellar motor, a role consistent with the presence of tetratricopeptide (TPR) repeats (protein–protein interaction motifs [29]) in these proteins. PflA has been tentatively assigned to the periplasmic medial disk, and PflB – to the cytoplasmic-membrane proximal disk, although the low resolution of the data precluded unambiguous determination of the exact locations of these proteins [21]. Based on the observation that the stator was not visible in the *pflA* and *pflB* mutants, it was suggested that in *C. jejuni*, the PflA/PflB complex serves as a scaffold that recruits stator complexes to the motor.

Disruption of the *pflA* gene in *H. pylori* also resulted in a flagellated but non-motile phenotype [30], in which the mutant cells displayed an altered structure at the base of the flagellum where the motor is located. This suggests that in *H. pylori*, PflA and PflB may also form part of the motor. However, the relatively low amino acid sequence identity to the *C. jejuni* counterparts (21% and 28% for PflA and PflB, respectively, Supplementary Figures S1 and S2) hints at the possibility of some differences in their biochemical properties, structural arrangement and function between *C. jejuni* and *H. pylori* and warrants an investigation into the nature and the implications of these differences.

Isolation of proteins that form periplasmic rings, such as PflA and PflB, has remained a challenge because these proteins often oligomerize via strongly hydrophobic regions that can drive aggregation when the protein is removed from its natural environment. Here, we report the isolation of the soluble forms of *H. pylori* PflA and PflB and the results of their biochemical characterization to support ongoing structural studies aimed at deciphering their location and their function within the *H. pylori* flagellar motor.

## Results

### Homology modelling-guided delineation of the domain boundaries and design of the expression constructs

**Figure 1:**
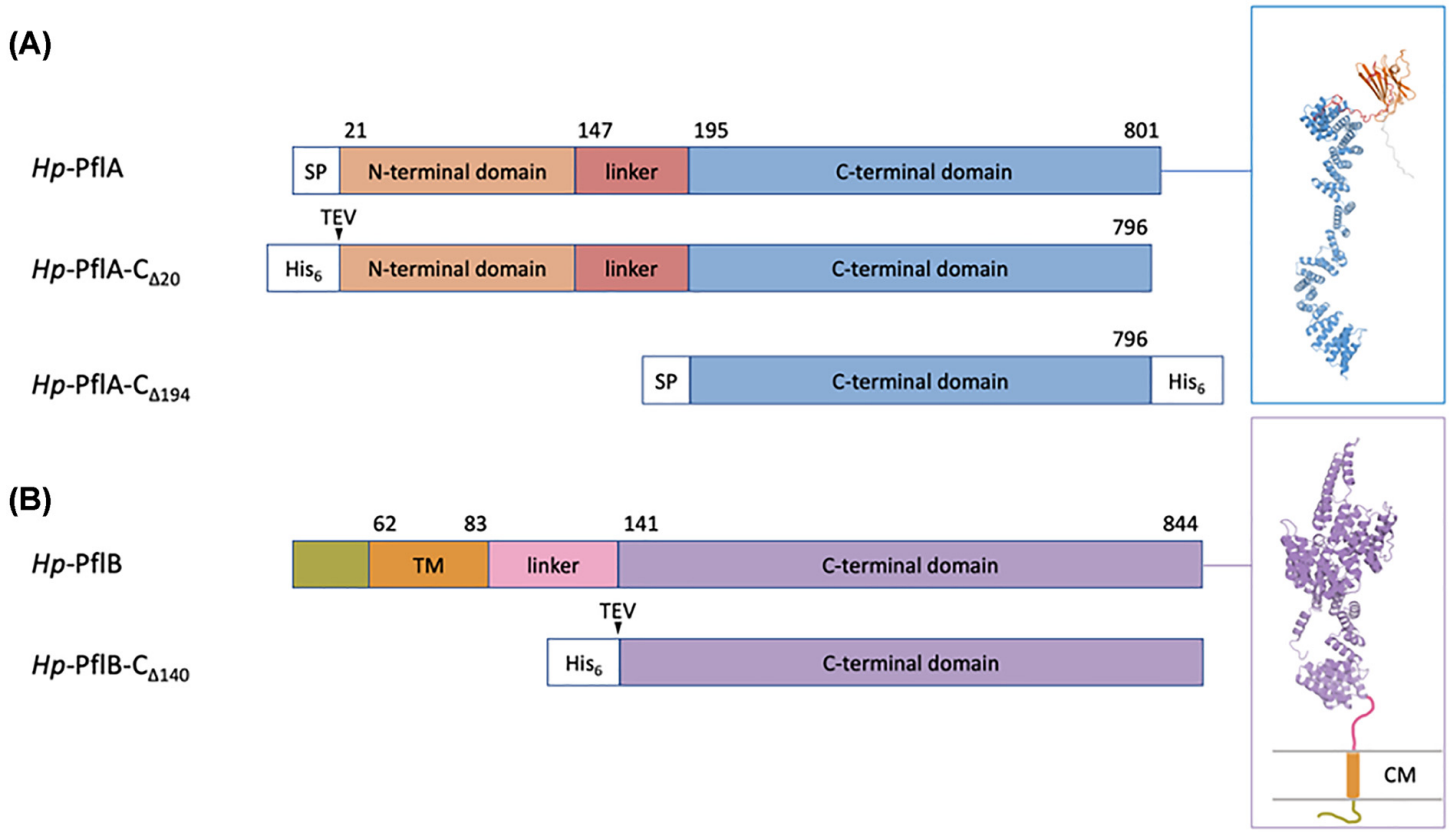
*H. pylori* PflA and PflB constructs used in this study (**A**) Schematics of native PflA and its PflA_Δ20_ and PflA_Δ194_ variants. SP: signal peptide. (**B**) Schematics of native PflB and its soluble variant PflB_Δ140_. TM: transmembrane helix; CM: cytoplasmic membrane.

Based on the amino acid sequence analysis, *H. pylori* PflA is predicted to be a periplasmic protein with a cleavable N-terminal signal peptide (amino acid residues 1-20). Analysis of its model structure generated by Alphafold2 (Figure 1) suggested that it contains two domains separated by a long, flexible linker: an N-terminal β-sandwich domain (residues 21-146), and a C-terminal TPR α-solenoid domain (residues 195-801). The five C-terminal residues of PflA (KNKES) were predicted to be disordered and could, therefore, impede crystallization in the future. To produce full-length PflA for structural studies (termed PflA_Δ20_), we therefore excluded the N-terminal signal peptide and the five C-terminal residues, and introduced a cleavable N-terminal His_6_ tag (Figure 1A).

β-rich domains can have aggregation propensities [31], which is why we also produced and tested a construct for expression of the C-terminal TPR domain only (PflA_Δ194_). To ensure that the expressed PflA_Δ194_ is correctly folded, we added a cleavable N-terminal signal peptide PelB that targets proteins for secretion into *E. coli* periplasm [32], and moved the His_6_-tag to the C-terminus [33] (Figure 1A).

*H. pylori* PflB is predicted to have a short cytoplasmic N-terminal region (61 residues), a single transmembrane helix (residues 62-83), that spans the bacterial inner membrane, and a large α-helical domain (residues 141-844) in the periplasm (Figure 1). The α-helical domain of PflB is connected to the transmembrane helix by an unstructured linker (84-140). Similar to the C-terminal domain of PflA, it contains TPR repeats. The construct for expression of the α-helical domain of PflB (PflB_Δ140_) was designed with a cleavable N-terminal His6-tag (Figure 1B).

### *H. pylori* PflA_Δ__20_ and PflB_Δ__140_ are expressed in *E. coli* as inclusion bodies but can be recovered using mild detergent sarkosyl

Overexpression of PflA_Δ20_ and PflB_Δ140_, assessed systematically under a range of test conditions (induction with 0–1 mM IPTG, temperature 289 K or 310 K) invariably resulted in the protein deposition in inclusion bodies (IBs). The expression conditions were therefore optimized to yield the highest levels of protein in IBs, which was achieved when the *E. coli* BL21(DE3) cells transformed with the respective plasmids were grown at 310 K, and the protein expression was induced with 0.1 mM IPTG for 4 h at the same temperature.

We first attempted to recover PflA_Δ20_ and PflB_Δ140_ from the IBs under denaturing conditions using a published procedure [34] that involved IB solubilization in a buffer containing 8 M urea, followed by protein refolding by dialysis, that removes urea, and affinity purification on an Ni-NTA column. However, this approach resulted in low recovery yield (∼4% for both proteins, Table 1). We could attribute this to (i) incomplete disruption of the IBs or (ii) formation of soluble aggregates, observable when either protein was subjected to size-exclusion chromatography (SEC), with most of the material eluting in the void volume (∼8 ml).

**Table 1:** Yield of PflA_Δ20_ and PflB_Δ140_ purification using refolding by dialysis

Refolding/purification step	PflA_Δ20_				PflB_Δ140_			
	Total protein (mg)	Step yield (%)	Overall yield (%)	Purity (%)	Total protein (mg)	Step yield (%)	Overall yield (%)	Purity (%)
Solubilization	60	100	100	70	55	100	100	70
Refolding	23	38	38	–	12	20	20	–
Ni-NTA	2.7	11	4	80	2.3	20	4	80

**Figure 2:**
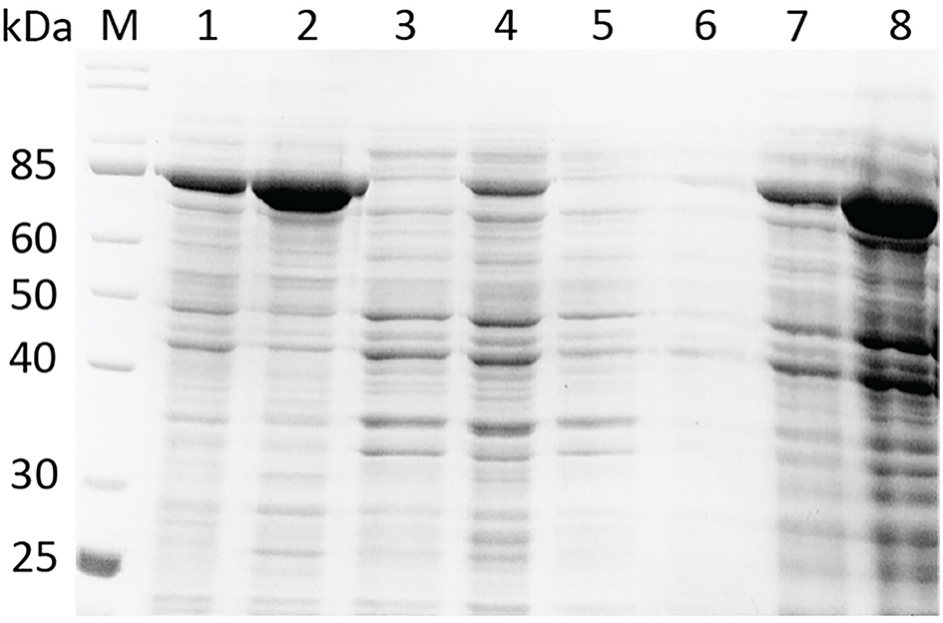
SDS-PAGE analysis of solubilization of PflA_Δ20_ inclusion bodies in buffers containing different additives The additives were (1) 8 M urea, (2) 8 M urea and sonication, (3) 2% DDM, (4) 2% LDAO, (5) 2% OG, (6) 0.5% sarkosyl, (7) 1% sarkosyl, (8) 3% sarkosyl. The solubilization screens were carried out by incubating 0.1 g IBs in 1 ml of the standard solubilization buffer (20 mM Tris-HCl pH 8.0, 150 mM NaCl) supplemented with urea or detergents. After solubilization, the samples were clarified by centrifugation, and the equal volume of supernatants were loaded on SDS-PAGE to assess the yield and purity.

To improve protein recovery from IBs, we tested a range of protocol modifications. We observed, for example, that sonicating IBs after their incubation in a 8 M urea buffer significantly improved the amount of PflA_Δ20_ in the supernatant (Figure 2, lane 2). We also hypothesized that, since PflA and PflB are thought to form rings in the periplasm, they may have hydrophobic regions that become exposed upon protein isolation, resulting in aggregation, and that the aggregation can be prevented by the addition of a mild detergent. We therefore performed solubilization screens in different detergents and established that the protein solubilization yield was the highest in the presence of 3% sarkosyl (Figure 2 lane 8 for PflA_Δ20_, data not shown for PflB_Δ140_).

### Purification screening revealed optimal conditions for efficient on-column refolding of PflA_Δ__20_ and PflB_Δ__140_

Next, we optimized the procedure for protein refolding by evaluating three different on-column refolding protocols. In the first protocol, proteins solubilized in a buffer containing 3% sarkosyl were loaded on to the Ni-NTA column, followed by a slow wash (2 h, 20 ml) of the loading buffer containing 0.05% n-Dodecyl-β-D-maltopyranoside (DDM), an overnight incubation at 277 K and elution with an elution buffer containing 0.05% DDM. The second protocol involved loading proteins solubilized in an 8 M urea buffer onto the column, washing with a loading buffer containing 0.05% DDM, an overnight incubation and elution with an elution buffer containing 0.05% DDM. The third protocol involved loading proteins solubilized in an 8 M urea buffer onto the column, washing with a loading buffers containing 3 M urea, then with a loading buffer with no urea, an overnight incubation at 277 K and elution with an elution buffer. The amount of protein adhered to the Ni-NTA column during the loading step was substantial (∼20 mg, starting from ∼60 mg of denatured IBs). The first protocol (solubilization in 3% sarkosyl, refolding in 0.05% DDM) demonstrated the highest on-column refolding yield (30% for PflA_Δ20_, PflB_Δ140_ similar) (Table 2).

**Table 2:** On-column refolding yield for PflA_Δ20_ (starting with 60 mg of denatured protein)

Additives in loading/elution buffer	3% sarcosyl/0.05% DDM	8 M urea/0.05% DDM	8 M urea/0 M urea
Adhered (mg)	20	21	19
Eluated (mg)	6.2	3.1	0.5
Yield (%)	30	15	2.5

### Size-exclusion chromatography analysis of PflA_Δ__20_ is consistent with presence of multiple conformations

**Figure 3:**
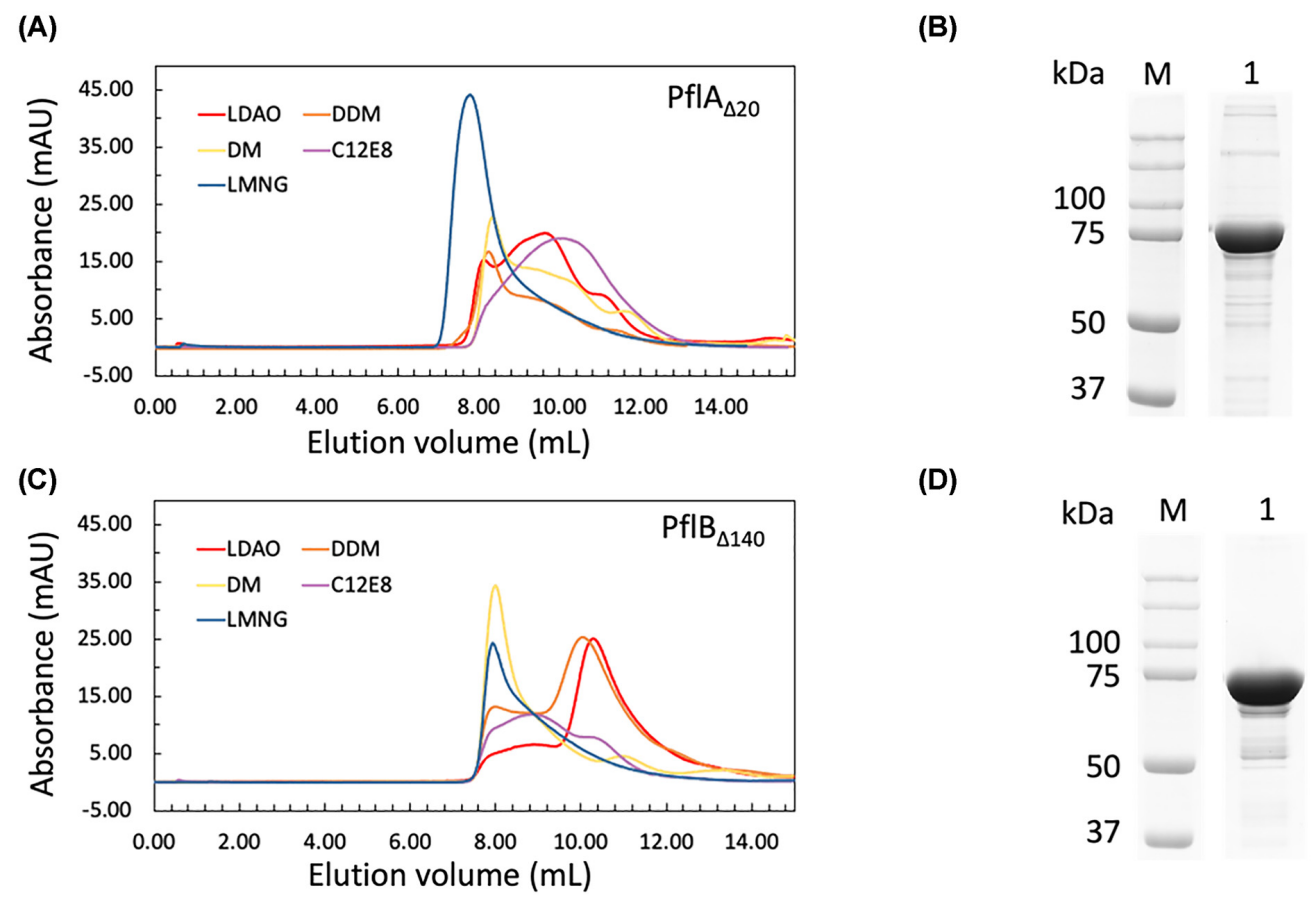
Size-exclusion chromatography analysis of PflA_Δ20_ and PflB_Δ140_ refolded and purified in different detergents (**A,C**) Elution profiles of LDAO-, DM- C12E8-, DDM-, and LMNG-purified PflA_Δ20_ and PflB_Δ140_ (**B,D**) Reduced SDS-PAGE analysis of the pooled SEC eluate illustrating the purity of PflA_Δ20_ and PflB_Δ140_ (15 μg) (the uncropped version of the gels is in Supplementary Figure S4).

To assess the behavior of PflA_Δ20_ in solution, we purified it in five different detergents (DDM, n-decyl-D-maltopyranoside [DM], n-dodecyl-N,N-dimethylamine-N-oxide [LDAO], octaethylene glycol monododecyl ether [C12E8] or lauryl maltose neopentyl glycol [LMNG]) using the first protocol for on-column (Ni-NTA) refolding followed by SEC. Purification in LMNG resulted in apparent aggregation, as most of the protein eluted in the void volume during the SEC step (Figure 3A). In contrast, the samples purified in DDM, DM, LDAO, C12E8 eluted before the void volume, but the elution profiles were very broad, extending from 8.3 to 13 ml, indicative of the presence of multiple different conformations. SDS-PAGE analysis demonstrated that the protein homogeneity of approximately 75% was achieved by following this procedure (Figure 3B shows LDAO-purified sample as an example). Starting with 3 mg of solubilized inclusion bodies, the protein yield was determined to be 0.84 ± 0.06 mg (mean ± SD) based on three independent replicates.

### Detergent screening identified conditions for production of monodisperse PflB_Δ140_

To characterize the behavior of PflB_Δ140_ in solution, we also purified it in the same five different detergents and compared the respective SEC elution patterns. The samples purified in LMNG and DM eluted close to the void volume (Figure 3C), suggesting that under those conditions, the protein forms soluble aggregates. In contrast, in DDM, LDAO and C12E8, the equilibrium shifted towards soluble oligomers. Notably, the major peak of the protein purified in LDAO (∼11 ml) was narrow and symmetric (Figure 3C), suggesting that the sample in LDAO was largely monodisperse. A PflB_Δ140_ monomer (MW 85 kDa) associated with the LDAO micelle (∼21.5 kDa) [35] would have an estimated MW of ∼115 kDa. The observed elution volume of ∼11 ml for PflB_Δ140_ in LDAO corresponds to a much larger complex with a molecular mass of approximately 417 kDa, indicating that PflB_Δ140_ forms at least a tetramer in the buffer with LDAO. SDS-PAGE analysis showed that the application of the described protocol allowed us to achieve PflB_Δ140_ homogeneity levels of approximately 78% (Figure 3C). The protein yield from 3 mg of solubilized inclusion bodies was 0.92 mg (±0.09 mg, *n*=3). It is important to note that one should not load more than 0.6 mg unfolded PflB_Δ140_ per mL Ni-NTA resin. We observed that exceeding this limit results in protein aggregation, with the LDAO-purified protein eluting in the void volume.

### SEC analysis suggested that PflA lacking β-rich N-terminal domain (PflA_Δ__194_) is monomeric in solution

**Figure 4:**
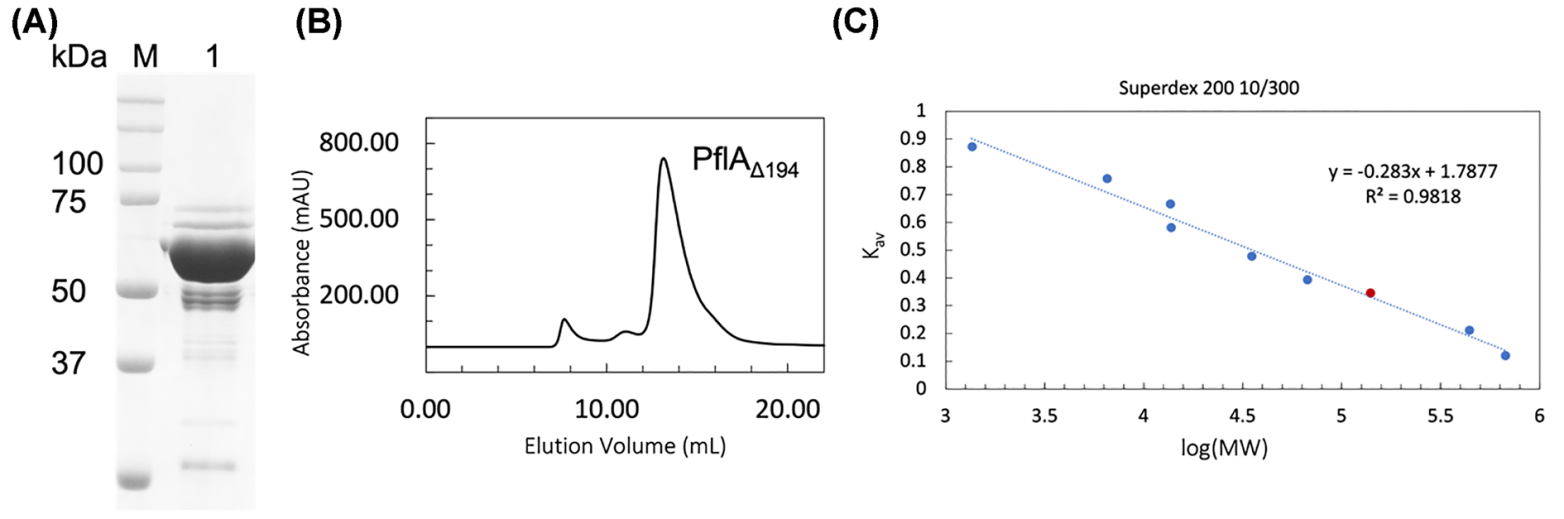
Size-exclusion chromatography analysis of PflA_Δ194_ (**A**) Reduced SDS-PAGE analysis of the pooled major SEC peak (15 μg of protein). (**B**) Elution profile of PflA_Δ194_ on Superdex 200 10/300 GL. (**C**) The SEC column calibration plot (*K*_av_ = *V*_retention_ − *V*_void_/*V*_column_ − *V*_void_) constructed using globular proteins of known mass. The data point for PflA_Δ194_ is in red.

PflA lacking the N-terminal domain (PflA_Δ194_) was expressed in a soluble form, allowing detergent-free purification using Ni-NTA affinity and SEC. The protein was purified to approximately 70% electrophoretic homogeneity based on Coomassie blue staining of the SDS-PAGE gel (Figure 4A). From 1 L of bacterial culture, 1.7 ± 0.5 mg of pure PflAΔ194 was obtained (mean ± SD, *n*=3). The cleavage of the signal peptide PelB has been verified by N-terminal sequencing of the purified protein. When subjected to SEC, most of PflA_Δ194_ eluted as a single, relatively narrow peak at a retention volume of ∼13.2 mL (Figure 4B). Estimation of the particle weight, based on the column calibration using globular proteins of known mass, yielded a value of approximately 122 kDa. Given that PflA_Δ194_ (MW 71 kDa) is predicted to have a very elongated structure (Figure 1A), it elutes earlier than expected for a globular particle of the same molecular weight. Our result, therefore, suggests that PflA_Δ194_ behaves as a monomer in solution.

### Circular dichroism (CD) analysis confirmed folded state of PflA_Δ__20_, PflB_Δ__140_ and PflA_Δ__194_

**Figure 5:**
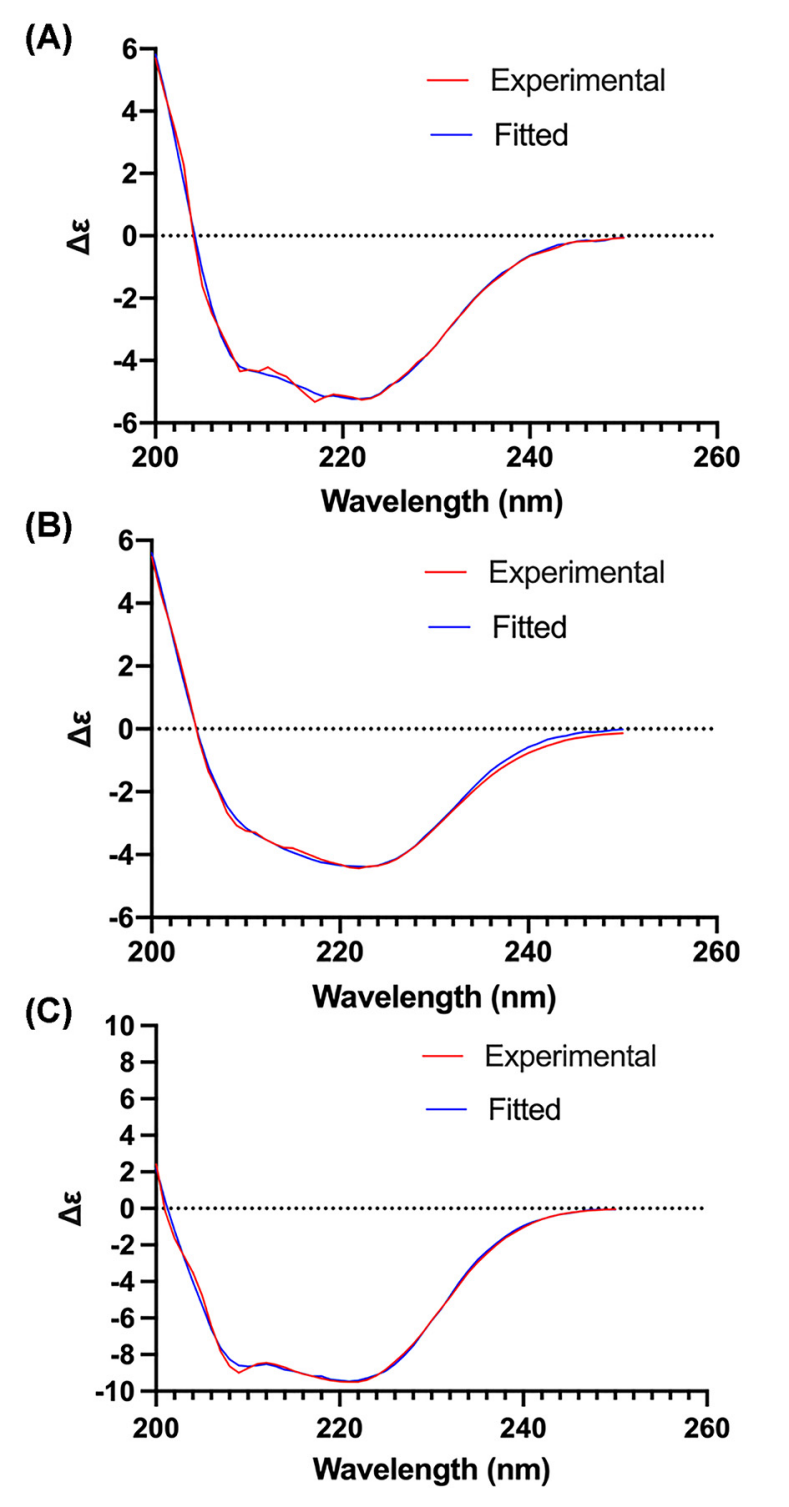
CD analysis of *H. pylori* PflA_Δ20_ (A), PflB_Δ140_ (B) and PflA_Δ194_ (C)

To ascertain structural integrity of PflA_Δ194_ and detergent-purified PflA_Δ20_ and PflB_Δ140_, we assessed their secondary structure using CD analysis (Figure 5). Estimation of the α-helix and β-sheet content for PflA_Δ20_ gave values (65% α and 10% β) that were close to those predicted by JPRED (68% α and 5% β) based on the analysis of its amino acid sequence, indicating that PflA_Δ20_ extracted from inclusion bodies is folded. Similarily, the CD-spectrum-derived secondary structure content of PflB_Δ140_ (64% α and 1% β) was close to that predicted from sequence analysis (79% α and 0% β), which confirmed the folded state of the protein. Finally, PflA lacking its N-terminal domain (PflA_Δ194_) was shown to be mainly α-helical, as expected, although the secondary structure content was lower than predicted (45% α and 3% β measured, 81% α and 0% β predicted). Together with the fact that PflA_Δ194_ is soluble, monomeric and monodisperse, this suggested that the protein is folded, but the removal of the N-terminal domain introduces intrinsic disorder in some parts of the protein.

## Discussion

In the present study, we developed a procedure for production of the soluble forms of *H. pylori* PflA and PflB which are believed to form part of the *H. pylori* flagellar motor. We showed that PflA_Δ20_ and PflA_Δ140_ are produced as inclusion bodies when expressed in *E. coli*, but they can be recovered using mild detergent sarkosyl. We devised an on-column refolding procedure for PflA_Δ20_ and PflB_Δ140_ that resulted in a higher protein yield than refolding by dialysis.

The SEC analysis of detergent-purified PflA_Δ20_ suggested the presence of multiple different conformations. This is consistent with the fact that PflA_Δ20_ is predicted to have two globular domains separated by a long flexible linker. In contrast, LDAO-purified PflB_Δ140_ behaved as a monodisperse species, indicating it has one predominant conformation. For both PflA_Δ20_ and PflB_Δ140_, the yield of soluble (non-aggregated) protein and sample monodispersity were highest in the LDAO buffer, suggesting these are the most optimal conditions for sample preparation for future biophysical studies.

We also showed that PflA lacking the β-rich N-terminal domain (PflA_Δ194_) is expressed in a soluble form rather than forming inclusion bodies. The PflA_Δ194_ construct corresponds to the solenoid C-terminal domain that contains 13 representative TPR motifs, likely involved in protein–protein interactions [36–39]. The soluble PflA_Δ194_ behaved as a monodisperse monomer in solution. However, the removal of N-terminal domain appeared to introduce intrinsic disorder in some parts of the protein, as evidenced by the reduction in α-helical content. The presence of the N-terminal signal peptide indicates that PflA is secreted into the periplasm via the SecYEG translocon, with the unfolded protein chain threaded through the secretion channel N-terminal domain first. Our observation that without the N-terminal domain, the C-terminal domain of PflA is partially destabilized lends support to the hypothesis that the N-terminal domain folds first in the preiplasm and acts as an intramolecular chaperone for the C-terminal domain.

The outcomes of this study are synergistic with recent progress in generating soluble forms of PflA and PflB from the closely related bacterium *Campylobacter jejuni* [40]. All *C. jejuni* constructs – PflA 16-788 (full length), PflA 169-788 (TPR regions), PflA 16-454 (N-terminal half) and PflB 113-820 – were expressed in *E. coli* and purified in a monomeric state, according to the results of the mass photomery assay. The methods for producing the soluble, folded forms of *H. pylori* PflA and PflB generated in this work will facilitate future biophysical and structural studies aimed at deciphering their location and their function within the *H. pylori* flagellar motor.

## Methods

### Reagents and bacterial strains

n-Dodecyl-β-D-maltopyranoside (DDM), n-decyl-D-maltopyranoside (DM), n-octyl-D-glucoside (OG), n-dodecyl-N,N-dimethylamine-N-oxide (LDAO), octaethylene glycol monododecyl ether (C12E8), and lauryl maltose neopentyl glycol (LMNG) (Supplementary Figure S3) were purchased from Anatrace. *Escherichia coli* BL21(DE3) strain was purchased from Novagen.

### Bioinformatics analysis

The amino acid sequences of PflA (UniProt ID A0A1Y3E2P7) and PflB (UniProt ID A0A1Y3E2Q1) from *H. pylori* strain SS1 were analyzed for the presence of transmembrane helices and signal peptides using the Phobius webserver [41]. Disordered regions were identified using the DISOPRED3 server (http://bioinf.cs.ucl.ac.uk/psipred) [42]. The secondary structure was predicted based on the amino acid sequence using the Jpred4 server (http://www.compbio.dundee.ac.uk/www-jpred/) [43]. The 3D structures were predicted using AlphaFold2 [44,45]. The figures showing 3D structures and sequence alignments were prepared using PyMol [Schrödinger, LLC. 2010] and ESPript [46], respectively.

### Cloning and overexpression of *H. pylori* PflA_Δ__20_, PflA_Δ__194_ and PflB_Δ__140_

The codon-optimized sequences encoding *H. pylori* PflA lacking the N-terminal signal peptide (PflA_Δ20_), and the periplasmic domain of PflB (PflB_Δ140_) were synthesized and ligated into the pET151/D-TOPO vector that adds an N-terminal TEV-cleavable His_6_-tag, by GenScript USA Inc (Figure 1 and Supplementary Material). The coding sequence for PflA lacking the N-terminal domain (PflA_Δ194_) was sub-cloned into the pET-22b (+) vector that adds a cleavable N-terminal periplasmic-targeting peptide PelB and a non-cleavable C-terminal His_6_-tag (Figure 1 and Supplementary Material). The expression constructs were confirmed by DNA sequencing. *E. coli* BL21(DE3) cells were transformed with the respective vectors, grown in LB medium containing 100 mg/ml ampicillin at 310 K until an OD_600_ of 0.8 was reached, at which point protein overexpression was induced by adding 0.1 mM IPTG, and growth was continued for a further 4 h at 310 K (for PflA_Δ20_ and PflB_Δ140_), or 16 h at 289 K for PflA_Δ194_. The cells were then harvested by centrifugation at 4,500 × ***g*** for 15 min at 277 K.

### Refolding and purification of *H. pylori* PflA_Δ__20_ and PflB_Δ__140_

For the on-column refolding and purification of PflA_Δ20_ or PflB_Δ140_, the cells were resuspended in buffer A (10 mM Tris-HCl pH 8.0, 100 mM NaCl) and lysed by sonication. The inclusion bodies (IBs) were pelleted by centrifugation at 10,000 × ***g*** for 15 min at 277 K, washed 3 times with (buffer A + 1% (v/v) Triton X-100) and 3 times with buffer A. The washed IBs were solubilized in buffer B (20 mM Tris-HCl pH 8.0, 150 mM NaCl, 10% (v/v) glycerol, 3% (w/v) sarkosyl) and incubated overnight with axial rotation at 293 K. The supernatant was cleared next day by centrifugation at 16,000 × ***g*** for 30 min, and diluted 3-fold (to 1% (w/v) sarkosyl [47]) with buffer C (20 mM Tris-HCl pH 8.0, 150 mM NaCl, 10% (v/v) glycerol, and 1% sarkosyl). About 3 mg of protein was loaded onto a 5 mL Ni-NTA column (GE Healthcare) (≤0.6 mg protein per ml resin [48]), equilibrated with buffer C. The column was washed with 3 column volumes (CVs) of buffer D (20 mM Tris-HCl pH 8.0, 150 mM NaCl, 10% glycerol, and 0.035% (w/v) DDM [equivalent to 4 times its critical micellar concentration (4 CMC)]) at the flow rate of 5 ml/min, then with 4 CVs of (buffer D + 10 mM imidazole) at the flow rate of 0.17 ml/min, and incubated overnight at 277 K. The protein was eluted with (buffer D + 500 mM imidazole), concentrated to 500 μl using a 100 kDa cut-off centrifugal filter and passed through the size-exclusion column Superdex 200 10/300 GL (GE Healthcare) equilibrated with buffer D. Detergent screening was performed by substituting DDM in all buffers with 4 CMC of DM, LDAO, C12E8 or LMNG (chemical structures of the detergents tested in this study are shown in Supplementary Figure S3).

The calibration curve for the size-exclusion column (*K*_av_ = −0.285 × Log(MW) + 1.79) was established by fitting the distribution coefficient (*K*_av_ = *V*_elution_ − *V*_void_/*V*_column −_ *V*_void_) and molecular weights (MW) of calibration standards listed in the manufacturer's manual to the equation *K*_av_ = A × Log(MW) + *B*. Protein concentration was determined using the Bradford assay [49], and the homogeneity of the purified protein was estimated from SDS-PAGE gel images using ImageLab software version 6.0.1.

### Purification of PflA_Δ__194_

The cells were resuspended in buffer E (20 mM Tris-HCl pH 8.0, 300 mM NaCl, 50 units/ml Pierce™ universal nuclease for cell lysis (Thermo Scientific, cat. No 88700), 1 mM MgCl_2_, 50 µM PMSF) and lysed by sonication. Cell debris were removed by centrifugation, NaCl and imidazole were added to final concentrations of 300 and 10 mM, respectively, and the sample was loaded on to a 5 ml Ni-NTA affinity column pre-equilibrated with buffer F (20 mM Tris-HCl pH 8.0, 300 mM NaCl) supplemented with 20 mM imidazole. The column was washed with 20 CVs of buffer F supplemented with 40 mM of imidazole, and PflA_Δ194_ was eluted with buffer F supplemented with 300 mM imidazole. The eluate was concentrated to 15 mg/ml using a 30 KDa cut-off centrifugal filter, and the sample was passed through the Superdex 200 10/300 GL column pre-equilibrated with buffer F (20 mM Tris-HCl pH 8.0, 300 mM NaCl). N-terminal sequencing of PflA_Δ194_ was conducted at the Monash University Biomedical Proteomics Facility.

### CD analysis

Far-UV CD spectra were recorded using a JASCO J600 spectropolarimeter. The spectra were collected over the wavelength range from 190 to 260 nm in a quartz cuvette with a 2-mm path length at a scan rate of 20 nm/min, and the results were averaged over 3 accumulated spectra. PflA_Δ20_ (0.03 mg/ml) and PflB_Δ140_ (0.05 mg/ml) were prepared and analysed in a buffer containing 20 mM Na phosphate buffer pH 7.5, 150 mM NaCl, 10% glycerol, and 4 CMC (0.092%) LDAO. PflA_Δ194_ (0.06 mg/ml) was analysed in a buffer containing 20 mM HEPES pH 7.5 and 300 mM NaCl. The secondary structure content was estimated from the CD spectra using the BeStSel server [50].

## Supplementary Material

Supplementary Figures S1-S4

## Data Availability

All supporting data are included within the main article and its supplementary files

## Open Access

Open access for this article was enabled by the participation of Monash University in an all-inclusive *Read & Publish* agreement with Portland Press and the Biochemical Society under a transformative agreement with CAUL.

## Abbreviations

CMC: critical micellar concentration
DDM: n-dodecyl-β-D-maltopyranoside
DM: n-decyl-D-maltopyranoside
IB: inclusion body
LDAO: n-dodecyl-N,N-dimethylamine-N-oxide
LMNG: lauryl maltose neopentyl glycol
OG: n-octyl-D-glucoside
PflA: paralyzed flagellum protein A
**PflB**: paralyzed flagellum protein B
SEC: size-exclusion chromatography
