# Supplementary material for: Biochemical characterization of paralyzed flagellum proteins A (PflA) and B (PflB) from Helicobacter pylori flagellar motor
Source: Biosci Rep. 2024 Sep 10;44(9):BSR20240692. doi: 10.1042/BSR20240692 (PMC11392913; doi:10.1042/BSR20240692)
Supplement: Supplementary Figures S1-S4 [file BSR-2024-0692_supp.pdf]

SUPPLEMENTARY DATA

Biochemical characterization of paralyzed flagellum proteins A (PflA) and B (PflB) from *Helicobacter pylori* flagellar motor

Xiaotian Zhou,[1] Yue Xin,[1] Kar L. Chan,[1] Anna Roujeinikova[1,2*]

[1]Department of Microbiology, Biomedicine Discovery Institute, Monash University, Melbourne, Victoria 3800, Australia

[2]Department of Biochemistry and Molecular Biology, Monash University, Melbourne, Victoria 3800, Australia

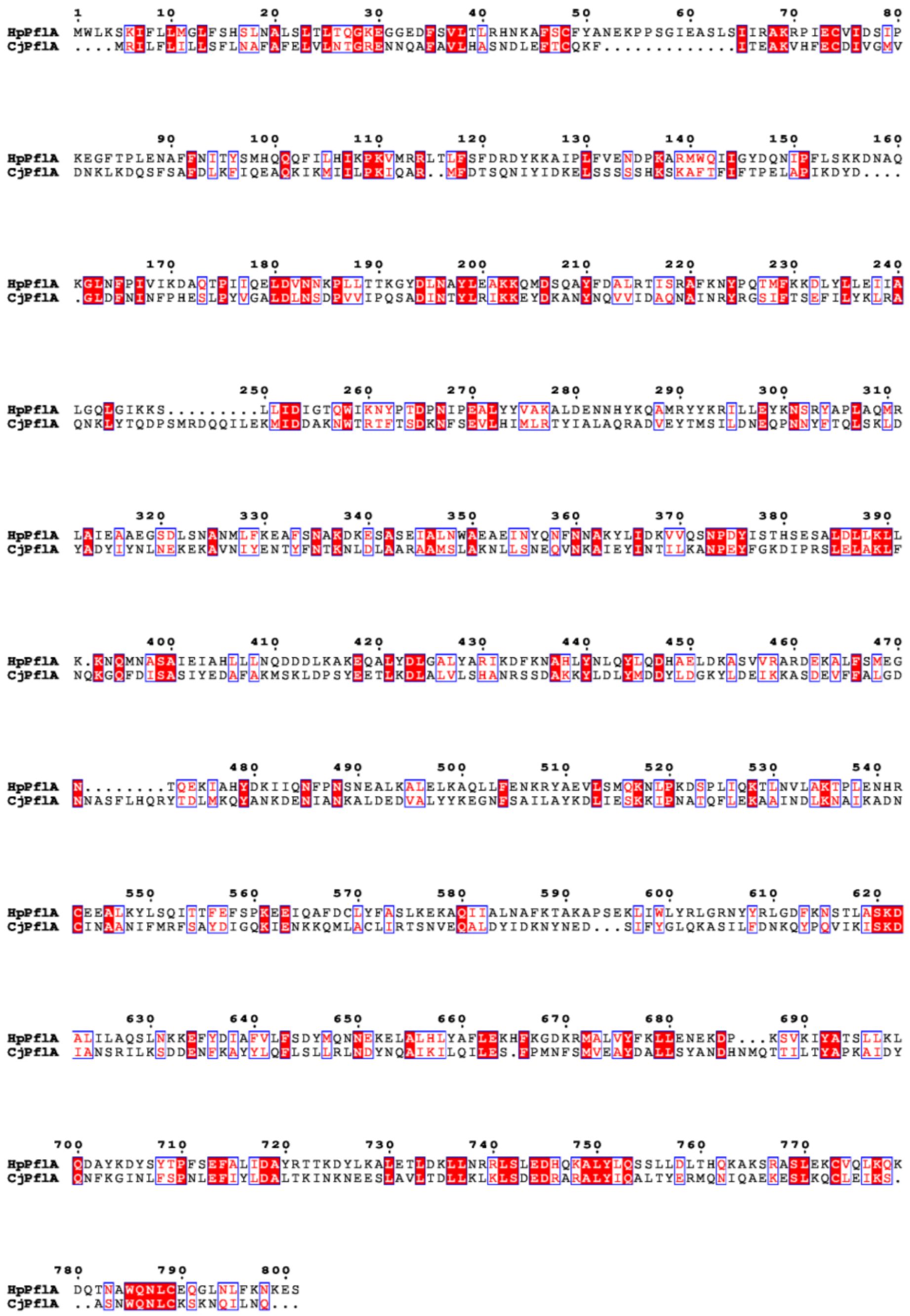

Supplementary Figure S1: **Sequence alignment of PflA from *H. pylori* (UniProt ID A0A1Y3E2P7) and *C. jejuni* (UniProt ID A0A0H3PAU5).**

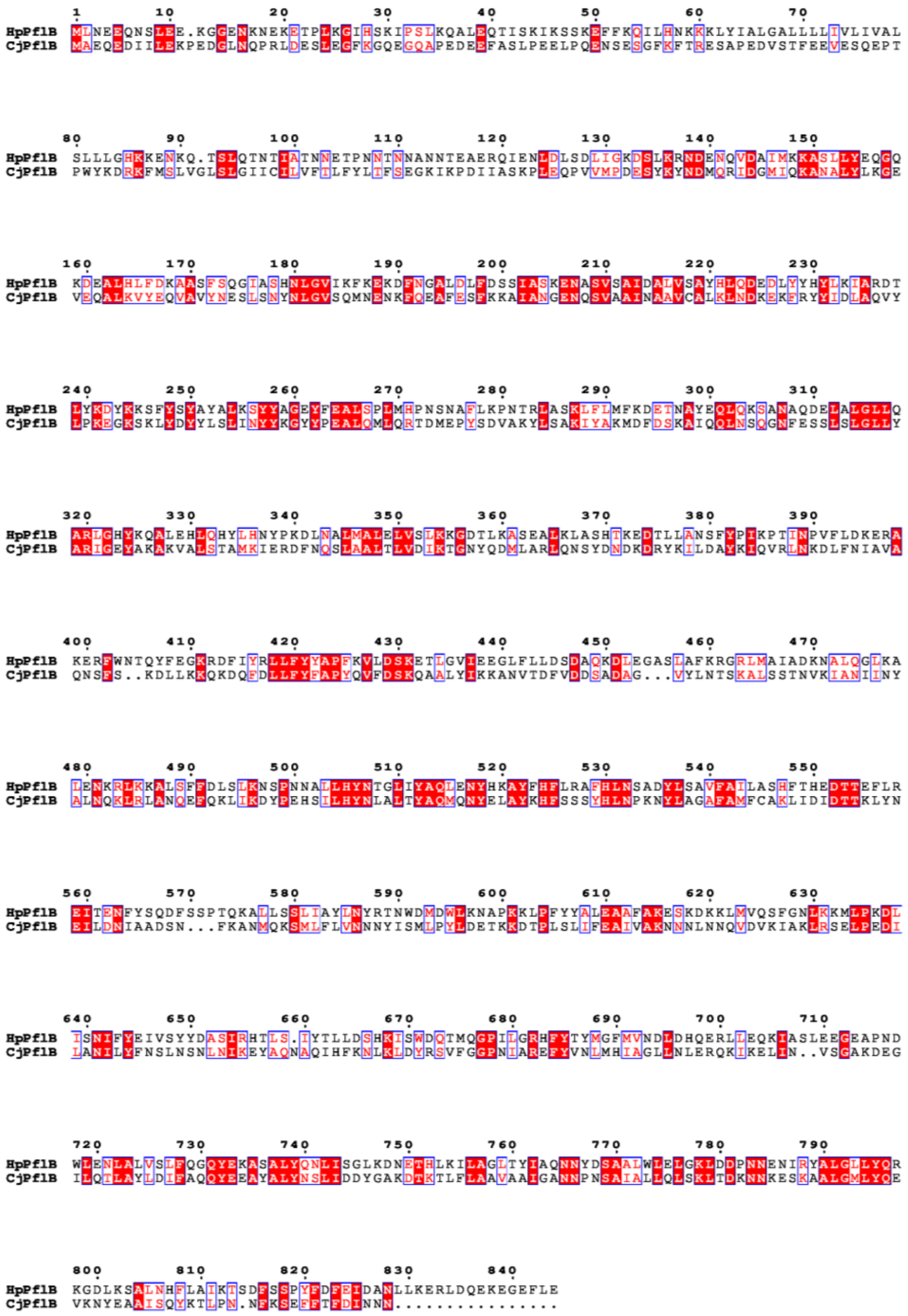

Supplementary Figure S2: **Sequence alignment of PflB from *H. pylori* (Uniprot ID A0A1Y3E2Q1) and *C. jejuni* (Uniprot ID A0A0H3PJ87).**

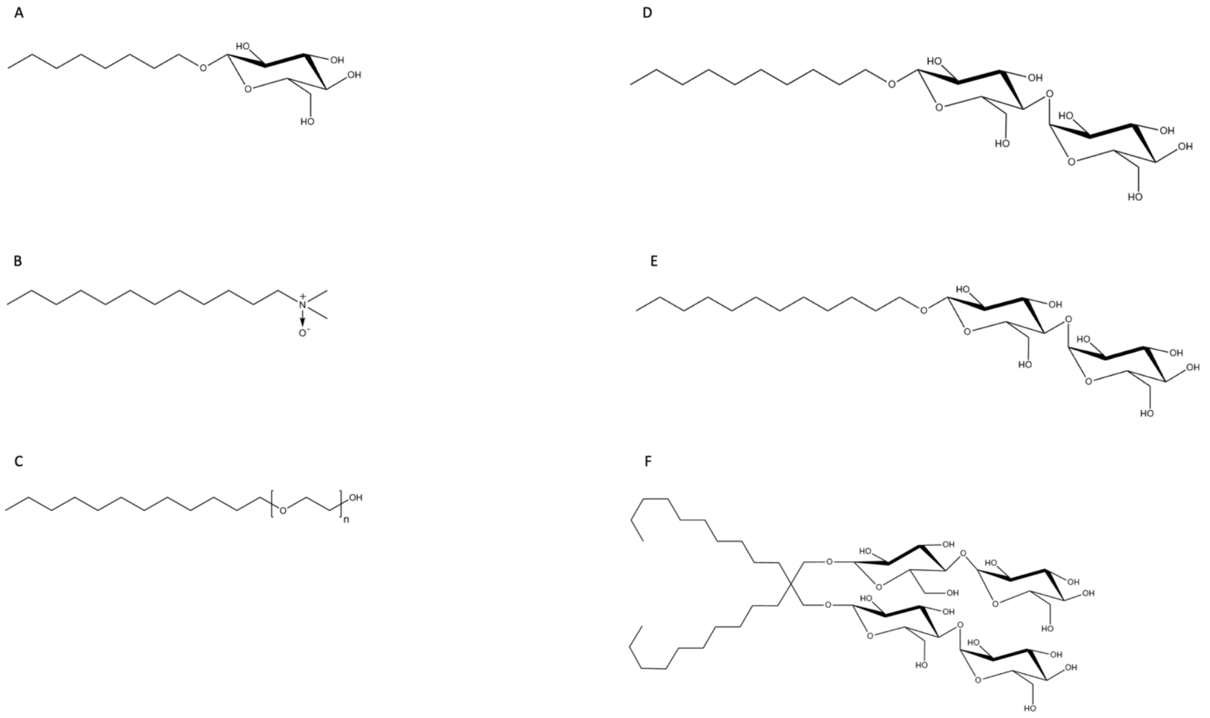

Supplementary Figure S3: **Chemical structures of the 6 detergents used in this study.**

A. Octyl glycoside (OG, $C_{14}H_{28}O_6$); B. Dodecyldimethylamine oxide (LDAO, $C_{14}H_{31}NO$); C. $C_{12}E_8$ ($(C_2H_4O)_nC_{12}H_{26}O$, n=8); D. Decyl maltoside (DM, $C_{22}H_{44}O_{11}$); E. Layrul maltoside (DDM, $C_{24}H_{46}O_{11}$); F. Lauryl maltose neopentyl glycol (LMNG, $C_{47}H_{88}O_{26}$).

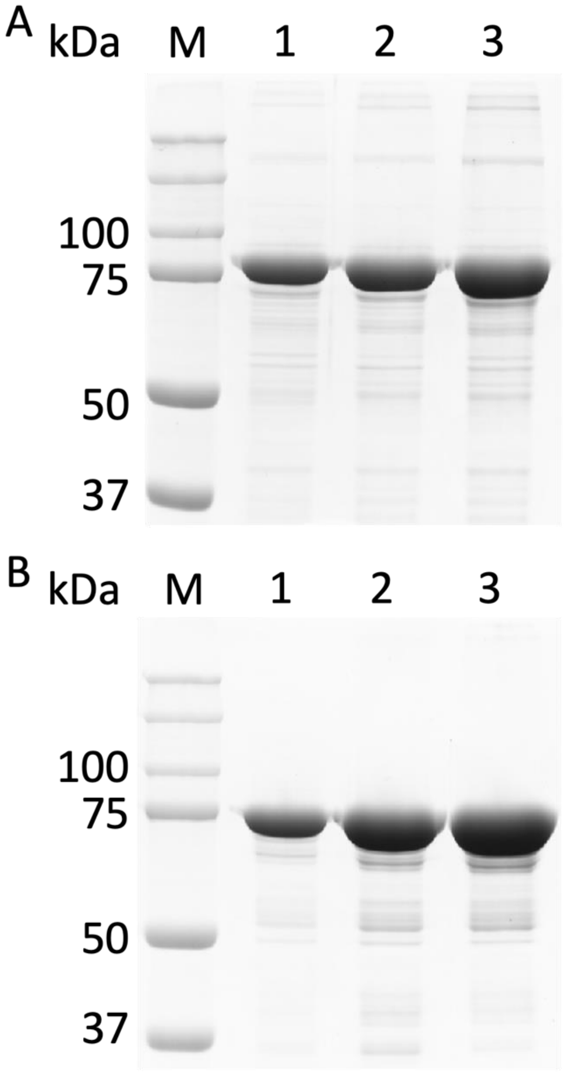

Supplementary Figure S4: **Reduced SDS-PAGE analysis of detergent-purified PflA$_{\Delta 20}$ and PflB$_{\Delta 140}$.** (A) M, protein marker; 1, PflA$_{\Delta 20}$ before Ni-NTA; 2. PflA$_{\Delta 20}$ after Ni-NTA; 3. PflA$_{\Delta 20}$ after SEC. Lanes M and 3 were used to prepare Figure 3 B. (B) M, protein marker; 1, PflB$_{\Delta 140}$ before Ni-NTA; 2. PflB$_{\Delta 140}$ after Ni-NTA; 3. PflB$_{\Delta 140}$ after SEC. Lanes M and 3 were used to prepare Figure 3 D.
